# Determinants of uncontrolled allergic rhinitis in Kinshasa hospitals

**DOI:** 10.3389/falgy.2023.1138537

**Published:** 2023-03-22

**Authors:** Patricia K. Kakobo, Hilaire K. Kalala, Joseph K. Kelekele, Paulin B. Mutombo, Dieudonné T. Nyembue, Peter W. Hellings, Jean-Marie N. Kayembe

**Affiliations:** ^1^ Department of Otorhinolaryngology, University of Kinshasa, Kinshasa, Democratic Republic of Congo; ^2^ Ophthalmology Department, University of Kinshasa, Kinshasa, Democratic Republic of Congo; ^3^ School of Public Health, University of Kinshasa, Kinshasa, Democratic Republic of Congo; ^4^ Department of Otorhinolaryngology-Head and Nose Surgery, University Hospital of Leuven, Leuven, Belgium; ^5^ Department of Otorhinolaryngology, Academic Medical Center, University of Amsterdam, Amsterdam, Netherlands; ^6^ Upper Airways Research Laboratory, University of Ghent, Ghent, Belgium; ^7^ Department of Pneumology, University of Kinshasa, Kinshasa, Democratic Republic of Congo

**Keywords:** uncontrolled allergic rhinitis, vitamin d, persistent form, moderate to severe form, Kinshasa

## Abstract

**Objective:**

To identify the determinants of uncontrolled allergic rhinitis (UCAR) in a hospital setting in Kinshasa, Democratic Republic of Congo.

**Methods:**

Hospital-based cross-sectional study of 153 patients with allergic rhinitis (AR). The diagnosis of AR was based on clinical grounds according to the Allergic Rhinitis and its Impact on Asthma (ARIA) criteria. Categorization into controlled AR (CAR) and UCAR was based on the visual analog scale (VAS with cut off point of 5). Binary logistic regression was used to identify factors associated with UCAR.

**Results:**

Patients with UCAR (60.1%) proportionally outnumbered those with CAR (39.9%). There were significantly more patients younger than 30 years of age among patients with UCAR. Factors significantly associated with UCAR were age below 30 years (OR = 3.31; 95% CI: 1.49–7.36; *p* = 0.003), low serum vitamin D level (OR = 3.86; 95% CI: 1.72–8.68; *p* = 0.001), persistent form (OR = 3.11; 95% CI: 1.39–6.98; *p* = 0.006) and moderate to severe form of AR (OR = 4.31; 95% CI: 1.77–10.49; *p* = 0.001).

**Conclusions:**

Factors associated with UCAR in this study population were younger age less than 30 years, low vitamin D level, and persistent as well as moderate to severe AR. Further studies are needed to elucidate the underlying mechanisms favoring the occurrence of these factors.

## Introduction

Allergic rhinitis (AR) is a disease of public concern, given its negative impact on patients' quality of life and socioeconomic power (1, 2). Poor control of AR is a leading cause of morbidity and mortality worldwide and accounts for 43% of the global disease burden (1). AR is generally underestimated, poorly controlled and undertreated (3). Although the symptoms of AR can be controlled with adequate treatment in most patients, recurrence is very common (3).

Currently, the most widely used tool to assess the severity and control of AR symptoms is the visual analog scale (VAS). Patients with a score ≥5 on this scale are considered to have uncontrolled AR (UCAR) (4, 5). Several studies have reported an increase in UCAR frequency in some countries such as France (71.7%) (6), Italy (>60%) (7), Tunisia (62%) (8), and the Democratic Republic of Congo (DRC) (75.5%) (9). On average, one-fifth of patients with AR have bothersome symptoms of AR despite adequate medical treatment abiding to the Allergic Rhinitis and its Impact on Asthma (ARIA) guidelines (10).

AR Treatment is aimed at controlling symptoms and risk factors for poor progression but also at improving the quality of life of patients. However, despite various recommendations for AR management, most patients remain inadequately controlled for several reasons, including noncompliance, comorbidities, misdiagnosis, and inadequate treatment. Poor control of AR can be caused by several factors such as asthma, rhinosinusitis, atopic dermatitis, and allergic conjunctivitis (11). A quick literature review indicates a clear lack of data on the risk factors for poor control of AR in sub-Saharan Africa (SSA) in general and in DRC in particular. The present multicenter study aims to determine the risk factors for poor control of AR and AR severity level in a hospital setting in Kinshasa.

### Patients and methods

The present study included patients with AR. It was conducted from November 2019 to May 2020 in otolaryngology departments of three Kinshasa hospitals, namely Cliniques Universitaires de Kinshasa (CUK), Centre Médical Diamant and Centre Hospitalier Monkole. The study was approved by the Biomedical Ethics Committee of the School of Public Health of the University of Kinshasa, abode to the guidelines of the Declaration of Helsinki, and all patients provided a written informed consent. *Patients were excluded for any of the following reasons: positive skin allergic test without any symptom of allergy; presence of allergic symptoms with a negative skin allergy test with un; pregnancy; current antihistamine treatment; any comorbidity or treatment affecting serum vitamin D level*.

### Diagnosis and classification of AR

The clinical diagnosis of AR was based on the ARIA classification and was confirmed by a positive allergen skin prick test (AST) (12). AR was then categorized into controlled allergic rhinitis (CAR) and UCAR based on the VAS in the last two weeks preceding the consultation. This categorization required AR patients to be adequately treated.

Patients scored their own symptoms on the VAS using a ruler graduated from 0 (total absence of symptoms) to 10 cm (maximum presence of symptoms) (12). Any patient with a score ≥5 was considered to have UCAR, whereas one with a score <5 was classified as having CAR (12). UCAR was intermittent if symptoms lasted less than 4 days/week and 4 weeks/year. On the other hand, it was persistent if symptoms lasted more than 4 days/week and 4 weeks/year (13). In addition, it was labelled as mild or moderate to severe depending on whether the symptoms were not very annoying or had an impact on quality (13).

### Allergic skin tests and serum vitamin D determination

The AST (Alyostal, Barcelona, Spain) consisted of a battery of nine allergens, namely dermatophagoides farinae, dermatophagoides pteronyssinus, blomia, 5-grasses, cat epithelium, dog epithelium, alternaria, aspergillus, and roach. The test was positive when the diameter of the skin papule induced by at least one allergen was equal to or greater than 3 millimeters, or equal to half the positive control (14).

25-hydroxyvitamin D3 was measured by radioimmunoassay using a Cobas E411 automatic well gamma counter (Roche Diagnostics International AG, Totkreuz, Switzerland) calibrated for iodine 125. For simplicity of analysis, serum vitamin D level was stratified into normal (≥30 ng/ml) and abnormal (<30 ng/ml).

Body mass index (BMI) was used to assess patients' nutritional status. Patients were further classified as underweight (BMI <18.5 Kg/m^2^), normal (BMI: 18.5–24.9 Kg/m^2^), overweight (BMI: 25–29.9 Kg/m^2^), and obese (BMI ≥30 Kg/m^2^) (15). The latter two groups were combined and analyzed as a single group.

### Statistical analysis

SPSS version 26.0 software was used for statistical analyses. Categorical variables were expressed as frequency and percentage, while quantitative variables were expressed as mean and standard deviation. Student's t-test was used to compare means of quantitative variables. Comparison of parameters of interest between patients with CAR and those with UCAR was performed using Pearson chi-square. Binary logistic regression was used to identify the determinants of UCAR. In the univariate model, gender, age groups, occupation, education level, residence (urban or semi rural), smoking (yes/no), BMI, number of allergens to which the patient is sensitized (mono vs. polysensitized), serum vitamin D level (normal vs. abnormal), allergic conjunctivitis (yes vs. no), asthma (yes vs. no), rhinosinusitis (yes vs. no), dermatitis (yes vs. no), high blood pressure (yes vs. no), number of people sharing the same room with the patient (≤2 vs. >2), use of an air conditioning system (yes/no), existence of pets (yes/no), presence of cockroaches in the house (yes/no), presence of trees and/or flowers in the house yard (yes/no), duration of illness (intermittent vs. persistent), and severity of illness (mild vs. moderate to severe) were used as predictors of AR control. Only variables that showed a significant association in the univariate model were analyzed in the multivariate model. The strength of association was estimated using the odds ratio (OR) at the p<0.05 significance level.

## Results

### Patients' sociodemographic and clinical characteristics

A total of 153 patients with AR were included in this study. The mean age was 32.1 ± 13.4 years for the whole group, 34.6 ± 13.1 years for patients with CAR, and 30.4 ± 13.3 years for those with UCAR. The other sociodemographic and clinical characteristics are shown in Table 1. Half of the patients were either under or at least 30 years old. Significantly more patients were female (62,7%), slightly more than half of the patients (54.9%) lived in urban areas, 56.9% reported a family history of atopy, and 69.3% had a university education. Most patients were sensitized to more than one allergen (60.8%), had a low serum vitamin D level (58.8%), shared the same bedroom with more than one other person (68.6%), and reported the existence of cockroaches in the house (64.7%) and trees in the yard (61.4%). Allergic conjunctivitis and rhinosinusitis were present in 52.9% and 69.3% of the patients, respectively. Patients with UCAR had a persistent form and a moderate to severe form of the disease in 69.3% and 75.2% of cases, respectively.

**Table 1:** Sociodemographic and clinical features in patients with controlled and uncontrolled allergic rhinitis.

Variables	Total	CAR	UCAR	Chi-square	*p*-value
	*n* = 153 (%)	*n* = 61 (%)	*n* = 92 (%)		
**Sex**					
Male	57 (37,3)	27 (44,3)	30 (32,6)	2,13	0,144
Female	96 (62,7)	34 (55,7)	62 (67,4)		
**Age range (years)**					
< 30	76 (49,7)	22 (36,1)	54 (58,7)	7,51	0,006
≥ 30	77 (50,3)	39 (63,9)	38 (41,3)		
**Occupations**					
Unemployed/Housewives	22 (14,4)	13 (21,3)	9 (9,8)	6,97	0,074
Paid occupations	60 (39,2)	25 (41,0)	35 (38,0)		
Tradesmen	20 (13,1)	9 (14,8)	11 (12,0)		
Students/Pupils	51 (33,3)	14 (23,0)	37 (40,2)		
**Level of study**					
Primary	6 (3,9)	4 (6,6)	2 (2,2)	5,54	0,063
Secondary	41 (26,8)	21 (34,4)	20 (21,7)		
University	106 (69,3)	36 (59,0)	70 (76,1)		
**Township of residence**					
Urban	84 (54,9)	31 (50,8)	53 (57,6)	0,32	0,574
Urban-rural	69 (45,1)	30 (49,2)	39 (42,4)		
**Smoking**	6 (3,9)	2 (3,3)	4 (4,3)	0,11	0,739
**AR in the family**	87 (56,9)	36 (59,0)	51 (55,4)	0,19	0,661
**BMI**					
Lean	16 (10,5)	4 (6,6)	12 (13,0)	1,67	0,433
Normal	66 (43,1)	27 (44,3)	39 (42,4)		
Overweight/Obesity	71 (46,4)	30 (49,2)	41 (44,6)		
**Number of allergens**					
Monosensitized	60 (39,2)	27 (44,3)	33 (35,9)	1,08	0,298
Polysensitized	93 (60,8)	34 (55,7)	59 (64,1)		
**Vitamin D level**					
Normal	63 (41,2)	33 (54,1)	30 (32,6)	6,99	0,008
Reduced	90 (58,8)	28 (45,9)	62 (67,4)		
**Allergic conjunctivitis**	81 (52,9)	25 (41,0)	56 (60,9)	5,82	0,016
**Asthma**	32 (20,9)	8 (13,1)	24 (26,1)	3,73	0,053
**Rhinosinusitis**	106 (69,3)	38 (62,3)	68 (73,9)	2,33	0,127
**Dermatitis**	52 (34,0)	18 (29,5)	34 (37,0)	0,91	0,341
**GERD**	55 (35,9)	20 (32,8)	35 (38,0)	0,44	0,507
**HBP**	25 (16,3)	13 (21,3)	12 (13,0)	1,83	0,176
**Number of people in the same bedroom**					
≤ 2	105 (68,6)	48 (78,7)	57 (62,0)	4,02	0,029
> 2	48 (31,4)	13 (21,3)	35 (38,0)		
**AC use**	56 (36,6)	18 (29,5)	38 (41,3)	2,20	0,138
**Domestic animals**	75 (49,0)	25 (41,0)	50 (54,3)	2,62	0,105
**Presence of cockroaches in the house**	99 (64,7)	35 (57,4)	64 (69,6)	2,38	0,122
**Trees in the parcel**	94 (61,4)	33 (54,1)	61 (66,3)	2,31	0,129
**ARIA classification**					
Intermittent	47 (30,7)	29 (47,5)	18 (19,6)	13,48	< 0,001
Persistent	106 (69,3)	32 (52,5)	74 (80,4)		
Mild	38 (24,8)	24 (39,3)	14 (15,2)	11,43	0,001
Moderate to severe	115 (75,2)	37 (60,7)	78 (84,8)		

AR, allergic rhinitis; BMI, body mass index; GERD, gastroesophageal reflux disease; HBP, high blood pressure; AC use, air conditioning use.

### Comparison of sociodemographic and clinical characteristics of patients with UCAR and CAR

Data in Table 1 also indicate that UCAR and CAR were present in 60.1% and 39.9% of patients, respectively. In patients younger than 30 years, UCAR was significantly more frequent than CAR (*p* = 0.006). There were significantly more patients with abnormal serum vitamin D levels among patients with UCAR than those with CAR (*p* = 0.008). A similar observation was made for patients with concomitant allergic conjunctivitis (*p* = 0.016). The persistent form (*p* < 0.001) and the moderate to severe form (*p* = 0.001) were also significantly more seen in patients with UCAR than in those with good AR control. Similarly, the proportion of patients who shared the same bedroom with more than 2 other people was significantly higher among those with poor than those with good AR control (*p* = 0.029).

### Factors associated with UCAR

We also sought to identify factors associated with poor control of AR. In univariate logistic regression (Table 2) including the sociodemographic and clinical variables listed in Table 1 as explanatory variables and the level of AR control (CAR vs. UCAR) as a dependent variable, age <30 years (*p* = 0.007), a low serum vitamin D level (*p* = 0, 009), sharing the same bedroom with more than 2 other people (*p* = 0.031), having concomitant allergic conjunctivitis (*p* = 0.017), permanent nature (*p* < 0.001), and moderate to severe severity of AR (*p* = 0.001) were significantly associated with UCAR. In the final multiple logistic regression model, only age <30 years, a low serum vitamin D level, permanent form, and moderate to severe form remained associated with UCAR. Specifically, patients younger than 30 years of age were 3.31 times more likely to have UCAR than those 30 years or older (*p* = 0.003). Based on serum vitamin D, those with a low serum vitamin D level had a 3.86-fold increased probability of having UCAR (*p* = 0.001). Similarly, patients with the permanent form and those with the moderate to severe form were 3.11 (*p* = 0.006) and 4.31 (*p* = 0.001) times more likely to have UCAR than those with the intermittent and mild forms, respectively.

**Table 2:** Factors associated with uncontrolled allergic rhinitis.

Variables	Univariate analysis			Multivariate analysis		
	Crude OR	CI 95%	*P*	Adjusted OR	CI 95%	*P*
Age (< 30 years)	2,52	1,29–4,91	0,007	3,31	1,49–7,36	0,003
Vitamin D level (abnormal)	2,44	1,25–4,74	0,009	3,86	1,72–8,68	0,001
Number of people in the bedroom (> 2)	2,27	1,08–4,77	0,031	1,92	0,79–4,62	0,148
Allergic conjunctivitis	2,24	1,16–4,33	0,017	2,15	0,99–4,66	0,053
Persistent allergic rhinitis	3,72	1,83–7,65	< 0,001	3,11	1,39–6,98	0,006
Moderate to severe allergic rhinitis	3,61	1,67–7,78	0,001	4,31	1,77–10,49	0,001

## Discussion

More than half (60.1%) of the patients interviewed in this study had a VAS score indicating poor control of AR. This frequency is similar to 60% reported in a multicenter study performed in non-asthmatic patients with AR symptoms in Italy (7) and 62% in another study in Tunisia (8). A higher frequency (71.7%) than ours was previously reported in France (6). On the contrary, the multinational study conducted in Egypt, Turkey and 3 countries of the Persian Gulf (Saudi Arabia, United Arab Emirates and Kuwait) reported an overall frequency of UCAR of 33% after assessment with the Rhinitis Control Assessment Test (RCAT). However, the frequency was higher in Egypt (55.6%) than in Turkey (27.9%) and in the 3 Persian Gulf countries combined (30.5%) (16). In China, an investigation in 250 AR patients prospectively assessed the frequency of UCAR using the Allergic Rhinitis Control Test (ARCT) at enrollment and then every 15 days after treatment and intensification of treatment in case of poor control. At enrollment, the incidence of UCAR was 99.2% before decreasing to 66% at 15 days, 29.2% at 30 days, 11.2% at 45 days, 3.6% at 60 days and 3.2% at 75 days after treatment (17). In Thai children, the Control of Allergic Rhinitis and Asthma Test (CARAT) showed a frequency of 28.2% in a hospital setting (18). In addition, a survey conducted in 5 European countries (Germany, Spain, France, Italy, and the United Kingdom) revealed, based on physicians' assessment, poor control of nasal AR symptoms in 18% and good control in 45.4% of patients regardless of the drug used (19). In Bousquet et al.'s study (20) on severe chronic upper respiratory disease, the incidence of UCAR after two weeks of treatment was 18% in patients treated based on physician's choice and 10.3% in those treated based on ARIA guidelines. Finally, in the AIMES survey conducted in 5 Middle Eastern countries (Egypt, Iran, Lebanon, Saudi Arabia, and the United Arab Emirates), 15% of respondents felt that their AR symptoms were poorly controlled compared to 40% whose symptoms were completely or well controlled despite taking medication to treat the symptoms (21). Several factors may contribute to the variability of UCAR frequency, including the type of study (cross-sectional vs. clinical trials), the type of instrument used to assess AR control, the characteristics of the study population, current or previous treatment, compliance with treatment, the level of knowledge and perception of the disease by the study population, and environmental factors. Ultimately, although the impact of treatment on AR control was not assessed in the present study, there is ample evidence to show that AR remains uncontrolled in a substantial number of patients despite well conducted treatment according to therapeutic guidelines (22). Despite the variability in the frequency of UCAR across studies and countries, the preceding data agree on the high frequency of UCAR.

We observed a significantly higher frequency of patients under 30 years of age among those with UCAR than those with CAR. Patients in this age group were 3.31 times more likely to have UCAR than those aged 30 years and older. *One possible explanation for this association is the lack, delayed or lack, refusal, delayed or inadequate treatment in young patients. In addition, medication high cost and the lack of health insurance prevent for most patients prevent them from being adequately treated.* Age is an important factor not only in awareness, but also in control of AR. In contrast, such an association was not found in the Italian multicenter study by Gani et al. (7). Other previous studies have described a strong association between allergic sensitization, asthma and rhinitis in children, adolescents, and young adults (23, 24). A separate analysis in the present series did not show a difference in the proportions of polysensitized between young (63.2%) and elderly (58.4%) subjects, *p* = 0.55. Elsewhere, investigations on allergic sensitization in different age groups consistently showed a biphasic trend of prevalence with age, with an initial increase until early adulthood and then a decrease (25, 26). Surprisingly, the prevalence of AR follows the same pattern (27, 28). This may suggest that patient's age plays a significant role in AR control. This hypothesis was tested in a prospective Korean study in which the clinical features of young (mean age: 28.9 ± 5.9 years) and elderly (mean age: 70.8 ± 5.4 years) AR patients were assessed before and after 4 weeks of treatment according to ARIA guidelines. Comparison of the Total Symptom Score (TSS), RCAT and VAS scores revealed that the therapeutic response was more favorable in young than in elderly patients on all assessment scales (29).

The association between AR and serum vitamin D level remains a controversial topic in light of conflicting results from different studies summarized in meta-analyses and reviews (30, 31). In the present study, however, we evaluated the association between vitamin D and the level of AR control in a cross-sectional manner. It is important to note that this aspect has been very rarely investigated. There were significantly more patients with low serum vitamin D levels among patients with UCAR than among those with CAR. The probability of having RANC was 3.86 times higher for patients with low serum vitamin D than for those with normal serum levels. A similar observation was made in two prospective studies evaluating the effect of vitamin D supplementation on the severity of AR. Kalsotra et al. evaluated the symptoms in two groups of patients with AR before and 4 weeks after administering oral vitamin D in combination with intranasal steroid sprays to one group and vitamin D alone to another group. After treatment, total nasal symptoms scores (TNSS) were significantly lower in both groups compared with pre-treatment scores, indicating an improvement in rhinitis symptoms and thus a progression towards control of AR (32). In another similar investigation, Modh et al. (33) evaluated two groups of 21 patients with AR and compared TNSS before and after routine antiallergic treatment and daily vitamin D supplementation for 21 days in one group and routine treatment only in the other. There was a significant post-treatment reduction in TNSS scores in both groups, but the reduction was significantly pronounced in the routine treatment only group. Similar results were reported in one more study including 35 cases and 33 controls with AR with similar serum vitamin D deficiency and nasal symptom severity scores. Eight weeks after treatment of cases with vitamin D plus a common anti-allergic (cetirizine) and controls with the common anti-allergic only, there was a significant increase in serum vitamin D levels in cases compared to controls in whom the level remained unchanged. There was also a significant difference between the nasal symptom scores of the two groups, mainly due to a significant reduction in scores in the cases (34). In summary, our observation and those of the studies listed above suggest that vitamin D deficiency is associated with poor control of AR.

It is noteworthy mentioning that there is a paucity of investigations on the association between control and severity as well as persistent or intermittent nature of AR. In the current series, poor control of AR was also independently associated with persistence and moderate to severe AR. This contrasts with findings from the Italian series where poor control of AR was not associated with disease duration (7). It is also important to underline that such an association described in a few studies was the result of confusion between poor control, severity, and response to AR treatment, probably stemming from the erroneous assumption that moderate to severe disease is uncontrolled. Indeed, in asthma, for example, where the relationship between severity and control has been extensively studied, it has been shown that the likelihood of a patient being controlled is not dependent on the severity of the disease before treatment (35, 36). Since the concept of control implies that patients are adequately treated beforehand (23), it cannot be excluded that the association found in the present study is rather a reflection of one or more of the factors such as lack of treatment, noncompliance in all its forms, application of a treatment regimen different from the ARIA guidelines, and treatment resistance.

Despite the fact that this study is multicentric and the first to systematically analyze the determinants of UCAR in the DRC, it has a number of limitations. First, the hospital-based, cross-sectional nature of the investigation and the small sample size (given the high prevalence of the disease in this setting) limit the generalizability of the results, and warrant the need for a larger, prospective study. *While we acknowledge that it would have been ideal to conduct a population-based study, it is also important to keep in mind that data from well conducted hospital-based studies are important as they may provide the first line of information about hospital utilization and basic epidemiologic measures needed for strategy planning, resource prioritization and allocation, and development of prevention, diagnosis, and management programs. In setting such as the DRC where population-based are difficult to conduct mainly due to limited funding, hospital data have become more valuable resources for studying epidemiology of diseases.* Second, the study population included some patients who were not adequately treated. As mentioned previously, including them may have influenced the reported results. Beyond these limitations, however, the present study has the merit of having investigated variations in serum vitamin D levels in relation to AR control using the reference tool and of having provided data suggesting that patients with UCAR are candidates for vitamin D supplementation. In addition, it has the merit of being considered as a first, to our knowledge, in sub-Saharan Africa addressing this issue.

In conclusion, this study shows that UCAR is frequent in the hospital environment of Kinshasa as previously reported. Age less than 30 years, vitamin D deficiency, permanent and moderate to severe nature of AR emerged as factors associated with UCAR in this series.

## Data Availability

The raw data supporting the conclusions of this article will be made available by the authors, without undue reservation.
